# Key immune cells and their crosstalk in the tumor microenvironment of bladder cancer: insights for innovative therapies

**DOI:** 10.37349/etat.2025.1002304

**Published:** 2025-03-31

**Authors:** Anna Di Spirito, Sahar Balkhi, Veronica Vivona, Lorenzo Mortara

**Affiliations:** Sun Yat-Sen University Cancer Center, China; Immunology and General Pathology Laboratory, Department of Biotechnology and Life Sciences, University of Insubria, 21100 Varese, Italy

**Keywords:** Bladder cancer, tumor microenvironment, immune evasion, immunotherapy

## Abstract

Bladder cancer (BC) is a heterogeneous disease associated with high mortality if not diagnosed early. BC is classified into non-muscle-invasive BC (NMIBC) and muscle-invasive BC (MIBC), with MIBC linked to poor systemic therapy response and high recurrence rates. Current treatments include transurethral resection with Bacillus Calmette-Guérin (BCG) therapy for NMIBC and radical cystectomy with chemotherapy and/or immunotherapy for MIBC. The tumor microenvironment (TME) plays a critical role in cancer progression, metastasis, and therapeutic efficacy. A comprehensive understanding of the TME’s complex interactions holds substantial translational significance for developing innovative treatments. The TME can contribute to therapeutic resistance, particularly in immune checkpoint inhibitor (ICI) therapies, where resistance arises from tumor-intrinsic changes or extrinsic TME factors. Recent advancements in immunotherapy highlight the importance of translational research to address these challenges. Strategies to overcome resistance focus on remodeling the TME to transform immunologically “cold” tumors, which lack immune cell infiltration, into “hot” tumors that respond better to immunotherapy. These strategies involve disrupting cancer-microenvironment interactions, inhibiting angiogenesis, and modulating immune components to enhance anti-tumor responses. Key mechanisms include cytokine involvement [e.g., interleukin-6 (IL-6)], phenotypic alterations in macrophages and natural killer (NK) cells, and the plasticity of cancer-associated fibroblasts (CAFs). Identifying potential therapeutic targets within the TME can improve outcomes for MIBC patients. This review emphasizes the TME’s complexity and its impact on guiding novel therapeutic approaches, offering hope for better survival in MIBC.

## Introduction

Cancer remains the second leading cause of death globally, with 2,001,140 new cases and 611,720 deaths projected in the United States alone in 2024. Among these, bladder cancer (BC) is estimated to account for approximately 83,190 new cases (63,070 in men and 20,120 in women) and 16,840 deaths (12,290 in men and 4,550 in women). Encouragingly, the incidence and mortality rates of BC have been declining in recent years. However, the persistent disparities in survival outcomes and the rising incidence rates of cancers such as breast, prostate, and melanoma underscore the urgent need for improved diagnostic and therapeutic strategies. Survival rates vary significantly, ranging from over 99% for localized prostate cancer to just 12% for advanced pancreatic cancer, highlighting the critical importance of prognostic biomarkers in guiding personalized care [1].

Over the years, cancer treatment has evolved dramatically, progressing from surgery and radiation to systemic chemotherapy, targeted therapies like trastuzumab, and immunotherapies such as chimeric antigen receptor T-cell (CAR-T) cells. While these advancements have significantly improved outcomes for certain cancers, challenges such as drug resistance, treatment-related toxicities, and limited efficacy in heterogeneous tumors persist. Emerging innovations, including circulating tumor DNA for early detection and therapies targeting oncogenic pathways, offer new hope. Continued investment in biomarker discovery and development will be essential for advancing personalized and effective cancer care [2].

BC is one of the most common cancers worldwide and presents a significant clinical and public health challenge [3]. It is the second most prevalent tumor of the urological tract, following prostate cancer in Europe and the United States [4]. BC can be further classified into non-muscle-invasive BC (NMIBC) and muscle-invasive BC (MIBC) [5].

Both environmental and genetic factors contribute to the development of BC. Environmental factors such as tobacco smoking and occupational exposures (e.g., in rubber production and firefighting) are well-established risk factors. Additionally, the International Agency for Research on Cancer (IARC) has identified other environmental exposures, such as X or gamma radiation and certain medications, as contributors to BC risk [6]. Chronic inflammation also plays a critical role in disease pathogenesis [7–9].

The tumor microenvironment (TME) plays a central role in regulating tumor initiation, progression, and therapeutic resistance [10]. The TME comprises tumor cells, infiltrating immune cells [e.g., macrophages, dendritic cells (DCs), and lymphocytes], cancer-associated stromal cells (e.g., cancer-associated fibroblasts, CAFs), cancer stem cells (CSCs), the extracellular matrix and numerous signaling molecules [11, 12]. Understanding the complexity of the TME is crucial for the development of effective anticancer therapies [10].

In BC, the complexity of the TME significantly contributes to therapeutic resistance through intricate cellular interactions, signaling pathways, and physical conditions. For instance, CAFs secrete growth factors, cytokines, and exosomes that activate key signaling pathways such as phosphoinositide 3-kinase (PI3K)-Akt, NF-κB, and STAT3 in tumor cells. These pathways reduce chemotherapy-induced DNA damage, suppress reactive oxygen species (ROS) production, and enhance cancer cell survival [13]. Immune cells within the TME, such as tumor-associated macrophages (TAMs) and myeloid-derived suppressor cells (MDSCs), further promote resistance by inhibiting cytotoxic T lymphocyte (CTL) and natural killer (NK) cells activity through the release of immunosuppressive cytokines, including interleukin-10 (IL-10) and transforming growth factor-beta (TGF-β). Abnormal tumor vasculature exacerbates these challenges by creating irregular, leaky, and poorly oxygenated blood vessels, which impede the delivery of therapeutic agents. This hypoxic and acidic environment fosters adaptive resistance mechanisms in tumor cells. Collectively, these factors make BC particularly resistant to treatment, posing significant therapeutic challenges [14].

Approximately 75% of newly diagnosed BCs are NMIBC, which are typically treated with transurethral resection followed by intravesical chemotherapy or Bacillus Calmette-Guérin (BCG) therapy [15]. In contrast, MIBC, an aggressive form of the disease, is generally managed with early radical cystectomy and pelvic lymph node dissection. Despite these therapeutic interventions, the 5-year cancer-specific mortality rate for BC remains high, underscoring the limitations of current treatment approaches [16].

Each subtype of BC exhibits distinct TME characteristics that influence its biological behavior and response to therapies. In NMIBC, the TME is generally less immunosuppressive compared to MIBC. The immune landscape in NMIBC is marked by a higher presence of effector immune cells, such as CD8^+^ CTLs, which play a crucial role in generating a robust anti-tumor response. This favorable immune environment contributes to the generally better prognosis observed in NMIBC patients. However, the TME in NMIBC can undergo dynamic changes, particularly in response to treatments like BCG therapy. Studies have shown that BCG can modulate the TME by enhancing immune cell infiltration and altering cytokine profiles, thereby boosting anti-tumor immunity. Despite these benefits, some NMIBC cases exhibit resistance to BCG, possibly due to the presence of immunosuppressive cells such as regulatory T cells (Tregs) and MDSCs, which can inhibit effective immune responses [3].

In contrast, MIBC is characterized by a more complex and immunosuppressive TME. This subtype often displays increased expression of inhibitory ligands, such as programmed death-ligand 1 (PD-L1), alongside a higher presence of immunosuppressive cells, including MDSCs and TAMs. These components contribute to an environment that suppresses anti-tumor immunity, thereby promoting tumor progression and metastasis. The immunosuppressive nature of the TME in MIBC presents significant challenges for immunotherapeutic interventions. However, recent studies have identified distinct TME subtypes within MIBC, characterized by varying levels of immune cell infiltration and stromal activity. These subtypes have been shown to predict responses to anti-PD-L1 therapies, suggesting that a deeper understanding of TME heterogeneity could lead to more personalized treatment strategies [17].

In this review, we delve into the key immune cell components of the TME, including CD8^+^ and CD4^+^ T cells, B cells, NK cells, TAMs, Tregs, MDSCs, neutrophils, CAFs and CSCs. Our aim is to provide a comprehensive understanding of the diverse cellular and molecular mechanisms that underlie immune cell responses in BC, including immune cell polarization and immune cell-TME crosstalk.

Understanding these mechanisms is crucial for advancing our knowledge of how the TME influences tumor progression and therapy responses, ultimately aiding in the development of more effective, individualized therapeutic strategies.

### The TME and BC

The TME plays a critical role in the development, progression, and metastasis of BC. It comprises a dynamic and complex network of cellular and molecular components, including immune cells, fibroblasts, endothelial cells, extracellular matrix proteins, and signaling molecules. These elements interact with tumor cells to create a supportive niche that facilitates cancer growth, promotes immune evasion, and contributes to therapy resistance [11].

Extracellular matrix remodeling in the TME is pivotal for immune cell infiltration, particularly involving T cells, macrophages, DCs, and neutrophils. The stiffness and density of the extracellular matrix create physical barriers that impede immune cell migration. Dense collagen regions, for example, exclude T cells, impairing their ability to perform immune surveillance and fostering tumor progression [18]. Beyond physical restrictions, extracellular matrix components act as damage-associated molecular patterns that activate immune cells through pattern recognition receptors. Molecules like biglycan and versikine, released during extracellular matrix degradation, stimulate macrophages and DCs to produce pro-inflammatory cytokines. However, this immune activation can also contribute to an immunosuppressive environment [19].

Macrophages, particularly TAMs, often polarize toward a pro-tumorigenic M2 phenotype, which promotes extracellular matrix deposition and remodeling, further hindering immune cell infiltration. Additionally, TAMs secrete proteases that degrade extracellular matrix components, creating tracks that facilitate tumor cell migration. Neutrophils, often recruited to hypoxic regions, contribute to extracellular matrix remodeling by releasing matrix metalloproteinases, enhancing angiogenesis and tumor progression [20]. The interplay between immune cells and extracellular matrix architecture dynamically influences the TME, often limiting effective immune responses. Targeting extracellular matrix remodeling could enhance immune cell infiltration and improve anti-tumor immunity by overcoming these physical and biochemical barriers.

Tregs and TAMs play significant roles in fostering an immunosuppressive microenvironment that promotes tumor progression and therapy resistance. Tregs exert immunosuppressive effects by releasing cytokines like IL-10 and TGF-β, which suppress the cytotoxic activity of effector T cells and NK cells. They also express elevated levels of immune checkpoint molecules, such as cytotoxic T-lymphocyte-associated protein 4 (CTLA-4) and PD-L1. These molecules inhibit T cell activation and proliferation, contributing to poor therapeutic outcomes and resistance to immune checkpoint inhibitors (ICIs) [21].

**Figure 1:**
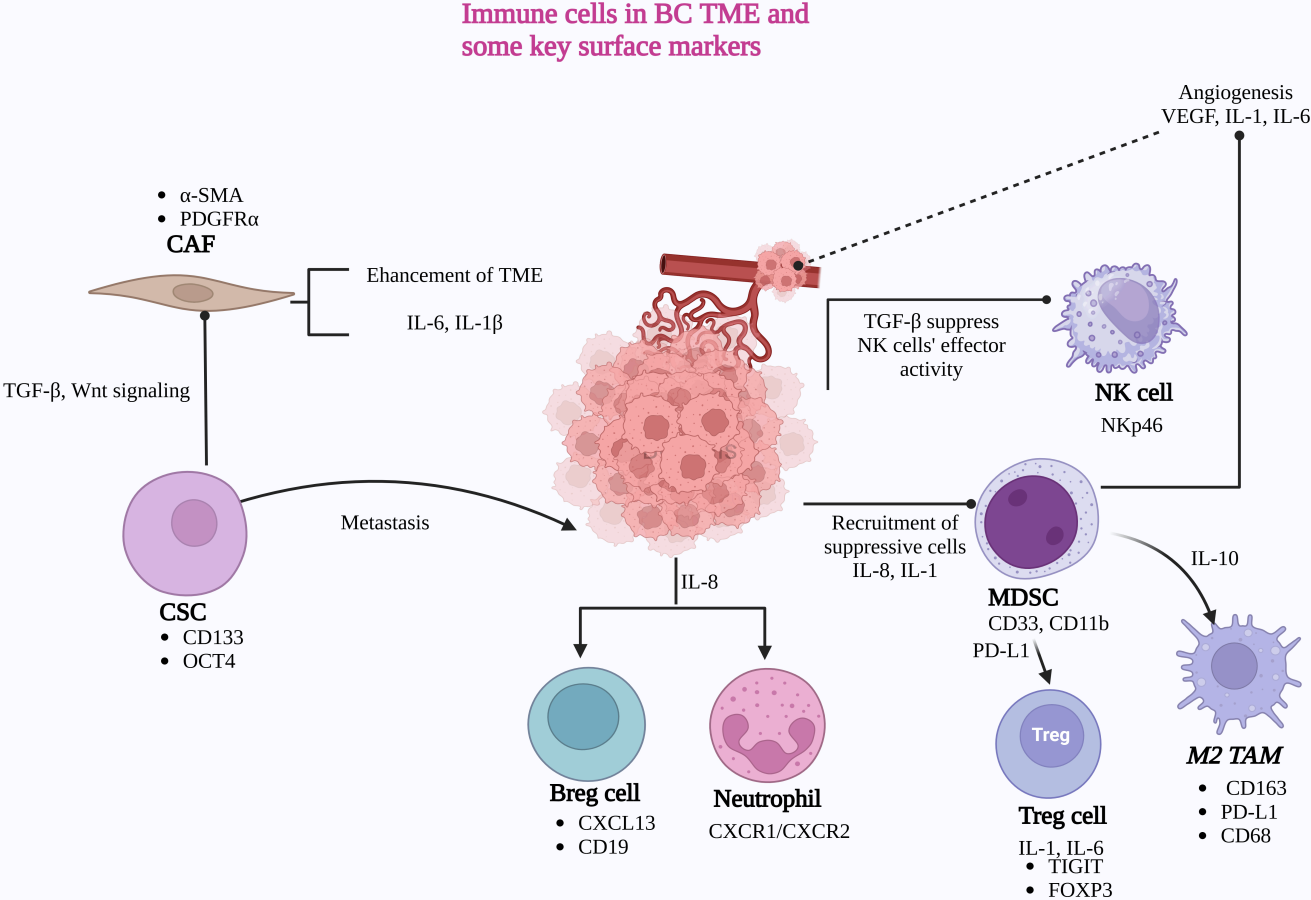
**The TME and its immune cell components with key surface markers**. CAF: cancer-associated fibroblast; TGF-β: transforming growth factor-beta; CSC: cancer stem cell; TME: tumor microenvironment; IL-6: interleukin-6; BC: bladder cancer; VEGF: vascular endothelial growth factor; NK: natural killer; MDSC: myeloid-derived suppressor cell; PD-L1: programmed death-ligand 1; TAM: tumor-associated macrophage; Breg: regulatory B cells; Treg: regulatory T cells. Created in BioRender. Balkhi, S. (2025) https://BioRender.com/a34h147

On the other hand, TAMs contribute to tumor progression by promoting pro-inflammatory signaling, angiogenesis, invasion, metastasis, and therapy resistance [22]. M2-type TAMs, in particular, amplify immunosuppression by releasing immunosuppressive cytokines, altering immune metabolism, and modulating immune checkpoint signaling pathways, such as programmed cell death protein-1 (PD-1)/PD-L1 signaling [23–25] (Figure 1).

Recent studies highlight the potential of urinary biomarkers and advanced sequencing technologies in understanding BC and its TME. Protein-based biomarkers like NMP22 and bladder tumor antigen have been explored extensively, but their limited sensitivity and specificity constrain their clinical utility. DNA-based biomarkers, such as those identifying mutations, methylation patterns, and microsatellite instability in urinary cell-free DNA, offer improved specificity. Similarly, RNA-based biomarkers, particularly non-coding RNAs like microRNAs, show promise in early detection and disease monitoring [26].

scRNA-seq has emerged as a valuable tool for transcriptional stratification, enabling the identification of distinct cell subpopulations and uncovering cellular heterogeneity in BC. Tan et al. [27] utilized scRNA-seq to develop a risk model with high prognostic and predictive efficacy for immunotherapy responses in BC. Their findings revealed increased immune cell infiltration and reduced therapeutic success in high-risk cohorts [27].

Additionally, Liu et al. [28] identified marker genes associated with exhausted CD8^+^ T cells using immune scRNA-seq data, shedding light on potential therapeutic targets to rejuvenate these cells. Exhausted CD8^+^ T cells represent a subset of immune cells present in the TME but are functionally impaired in eradicating cancer cells [29].

Insulin-like growth factor-binding proteins have also been implicated in TME dynamics. For example, IGFBP2 and IGFBP5 have been identified as prognostic markers in gliomas and urothelial carcinomas, respectively. Their overexpression correlates with tumor aggressiveness, immune evasion, and poor patient outcomes [30, 31]. Additionally, insulin-like growth factor-binding proteins are associated with immune cell infiltration and response to therapies, underscoring their potential as therapeutic targets or predictive biomarkers.

A comprehensive understanding of the TME is essential to uncover the mechanisms driving BC progression and treatment resistance. By unraveling the interplay between tumor cells and their microenvironment, researchers can identify novel therapeutic targets and develop innovative treatment strategies. Advancements in technologies like scRNA-seq and biomarker discovery pave the way for improved cancer prognostics and personalized therapies. In the following sections, we will delve deeper into the components of the TME, exploring their specific roles in BC progression and their potential for therapeutic intervention (Figure 1).

The TME is composed of a variety of immune and stromal cells, including CAFs, CSCs, regulatory B cells (Bregs), neutrophils, Tregs, M2-TAMs, MDSCs, and NK cells. These cells contribute to tumor progression and immune modulation in the TME. CSCs promote tumor metastasis and release TGF-β, which activates CAFs through TGF-β and Wnt signaling pathways. Once activated, CAFs enhance the immunosuppressive TME by secreting IL-6 and IL-1β. Tregs create an anti-inflammatory environment by releasing TGF-β and IL-10, suppressing the cytotoxic activity of NK cells. Additionally, IL-8 and IL-1 recruit neutrophils and suppressive immune cells, including TAMs and MDSCs, further promoting immune evasion and angiogenesis through the secretion of vascular endothelial growth factor (VEGF), IL-1, and IL-6.

Key surface markers involved in these immune processes include NKp46 on NK cells, which enhances their cytotoxic activity. CD163 and PD-L1 on M2-TAMs facilitate immunosuppression by promoting anti-inflammatory responses and inhibiting T-cell activity. CD33 and CD11b on MDSCs are associated with their immunosuppressive phenotype, which helps in inhibiting T-cell responses and promotes tumor immune evasion. TIGIT and FOXP3 on Tregs enhance their immunosuppressive functions by inhibiting effector T-cell responses and maintaining immune tolerance. CXCR1 and CXCR2 on neutrophils mediate their recruitment and activation, driving pro-tumorigenic functions. CXCL13 and CD19 on Bregs contribute to immune suppression by supporting regulatory functions that inhibit anti-tumor immune responses. CD133 and OCT4 on CSCs are key markers related to self-renewal, tumor initiation, and therapy resistance, promoting cancer progression and recurrence. Lastly, α-SMA and PDGFRα on CAFs play roles in tumor progression by driving extracellular matrix remodeling, fibrosis, and paracrine signaling that supports cancer cell growth and immune evasion.

## Cancer-associated fibroblasts (CAFs)

CAFs represent a critical component of the TME, playing diverse roles in cancer progression, immune evasion, and therapy resistance. Fibroblasts, the most abundant cell types in connective tissue, primarily arise from a mesenchymal origin. However, they may also originate from mesothelial-, epithelial-, or endothelial-to-mesenchymal transitions or hematopoietic cells. Despite their varied origins, fibroblasts lack epithelial, endothelial, and leukocyte markers [32].

In the bladder microenvironment, tissue fibroblasts reside in the sub-urothelial lamina propria, constructing the extracellular matrix and transmitting signals during physiological bladder filling or pathological stress [33]. Under homeostatic conditions, fibroblasts maintain stromal architecture and function, remodeling the extracellular matrix, promoting angiogenesis, releasing growth factors, aiding in wound healing, and collaborating with immune cells [34]. Their involvement in wound healing includes resolving inflammation by facilitating crosstalk with epithelial and immune cells [35] (Table 1).

**Table 1:** Overview of some important immune cell types, their roles in the TME of BC, associated markers, and therapeutic implications

Immune cell type	Role in BC TME	Markers	Therapeutic implications
Tumor-associated macrophages (TAMs)	TAMs are abundant in the TME and polarize into pro-tumorigenic M2 macrophages, promoting immunosuppression, angiogenesis, and tumor progression.	CD68, CD163 (M2), iNOS (M1), ARG1	Reprogramming TAMs from M2 to M1 using cytokines or inhibitors can enhance anti-tumor immunity. Targeting PD-L1 on TAMs or disrupting recruitment pathways (e.g., CCL2-CCR2) is under investigation
Myeloid-derived suppressor cells (MDSCs)	MDSCs suppress T cell activation, inhibit immune responses, and create a microenvironment favoring tumor progression.	CD11b, CD33, CD15, CD14	Targeting MDSCs with agents that inhibit recruitment or function can improve immune checkpoint blockade efficacy
Regulatory T cells (Tregs)	Tregs suppress immune responses by inhibiting cytotoxic T cells and natural killer cells, contributing to immune escape.	CD4, CD25, FOXP3, TIGIT	Reducing Treg activity or targeting TIGIT can enhance anti-tumor immunity
Cytotoxic T lymphocytes (CTLs)	CTLs mediate anti-tumor effects by recognizing and killing tumor cells. In BC, their activity is often impaired due to TME immunosuppression.	CD8, granzyme B, perforin	Immune checkpoint inhibitors targeting PD-1/PD-L1 restore CTL functionality and enhance tumor cell killing
Natural killer (NK) cells	NK cells can kill tumor cells directly and influence the TME through cytokine production.	CD56, CD16, NKp46	Activating NK cells or enhancing their cytotoxicity through cytokines or immune modulators can improve anti-tumor responses
Dendritic cells (DCs)	DCs are crucial for antigen presentation and the activation of T cells. However, in the TME, their functionality is often impaired.	CD11c, HLA-DR, CD80/CD86	Therapies to enhance DC activation or antigen presentation are being explored, including DC-based vaccines
B cells	B cells exhibit dual roles, promoting or inhibiting tumor progression depending on subtype. They are involved in antibody production and modulating immune responses.	CD19, CD20, CD138, CXCL13	Targeting immunosuppressive regulatory B cells (Bregs) or enhancing tertiary lymphoid structures (TLSs) can improve anti-tumor immunity
Cancer stem cells (CSCs)	CSCs contribute to tumor recurrence, therapy resistance, and immune evasion by interacting with the TME.	CD44, CD133, OCT4, SOX2	Targeting CSCs with therapies directed at their unique markers and pathways (e.g., Wnt/β-catenin, STAT3) may reduce recurrence and enhance treatment efficacy
Cancer-associated fibroblasts (CAFs)	CAFs remodel the extracellular matrix, promote immune evasion, and secrete cytokines that suppress T cell activity.	α-SMA, FAP, PDGFRα/β, CXCL12	Modulating CAF activity or targeting pathways like TGF-β signaling can enhance immune cell infiltration and therapy responsiveness
Neutrophils	Neutrophils release enzymes that remodel the extracellular matrix and promote angiogenesis and metastasis.	CD66b, MPO, CXCR1/CXCR2	Targeting tumor-associated neutrophils (TANs) or their recruitment pathways (e.g., CXCR1/2) may reduce metastasis and enhance anti-tumor responses

TME: tumor microenvironment; iNOS: inducible nitric oxide synthase; PD-L1: programmed death-ligand 1; BC: bladder cancer; PD-1: programmed cell death protein-1; SOX2: SRY homology box 2; FAP: fibronectin attachment protein; TGF-β: transforming growth factor-beta

However, cancer cells subvert normal fibroblast functions, exploiting them to evade immune surveillance, sustain proliferation, and invade surrounding tissues. Chronic inflammation, a hallmark of tumorigenesis, influences stromal and immune cells, fostering a pro-tumorigenic environment [36]. Fibroblasts exposed to persistent inflammatory signals transition into CAFs, becoming permanently activated. CAFs act as synthetic machines for tumor tissue components [37].

CAFs can originate from resident fibroblasts, mesenchymal stem cells, pericytes, adipocytes, or epithelial and endothelial cells undergoing transdifferentiation [38–41]. They exhibit an elongated shape, express mesenchymal markers like vimentin, α-smooth muscle actin, fibroblast activation protein, fibroblast-specific protein 1, desmin, and platelet-derived growth factor receptors, while remaining negative for epithelial, endothelial, and leukocyte markers [40].

Within the TME, CAFs facilitate immune evasion by remodeling the extracellular matrix, creating physical barriers that hinder drug efficacy and immune cell infiltration. CAFs secrete cytokines and other factors that upregulate immune checkpoint molecules, further suppressing immune responses. For instance, research has shown that CAF-derived CXCL12 enhances immune escape mechanisms in BC by regulating PD-L1 expression and promoting tumor cell proliferation, invasion, and migration [42, 43].

scRNA-seq has revealed CAF heterogeneity, identifying subpopulations with distinct functions in BC progression. Notably, a subset characterized by PDGFRα^+^ITGA11^+^ markers correlate with lymphovascular invasion (LVI) and poor outcomes. These CAFs promote lymphangiogenesis through interactions with lymphatic endothelial cells, activating the SRC-p-VEGFR3-MAPK pathway. Additionally, they secrete CHI3L1, remodeling the extracellular matrix to aid in cancer cell intravasation and lymphatic metastasis [44].

Inflammatory signals, such as IL-1 via the NF-κB pathway and IL-6 via STAT transcription factors, are key activators of CAFs [45, 46]. IL-1β-enriched conditioned media from CAFs has been shown to activate Wnt signaling, promoting BC proliferation and metastasis [47]. Similarly, IL-6 is linked to genomic stress and worse prognoses in patients with high serum levels [48, 49]. Physical stress from hyperproliferative cancer cells and extracellular matrix alterations also activates transcriptional regulators like heat shock factor-1, further driving CAF formation [50–53].

In BC, four CAF subpopulations have been identified:

- A resting fibroblast-like subtype.
- An inflammatory CAF (iCAF)-like phenotype expressing IL-6, CXCL12, and CXCL2.
- A myofibroblastic CAF (myCAF)-like subtype marked by extracellular matrix and focal adhesion molecule expression.
- Interferon (IFN)-regulated CAFs, which express growth factor-related genes (e.g., *NRG1*, *STC1*, and *WNT5A*) [54–56].

MyCAFs and iCAFs play immunomodulatory roles. MyCAFs contribute to an immune-cold microenvironment by creating dense extracellular matrix that restricts CD8^+^ T cell infiltration. Conversely, iCAFs secrete inflammatory cytokines like IL-6 and CXCL12, driving tumor cell proliferation, angiogenesis, and immune escape [40]. IL-6, particularly from iCAFs, has been implicated in transforming non-invasive BC cells into invasive phenotypes and correlates with poor patient outcomes [49, 56].

Emerging evidence highlights additional CAF mechanisms, such as macrophage-myofibroblast transition (MMT), wherein M2-polarized macrophages transition into CAF-like cells via Smad3 activation. Suppressing Smad3 in macrophages reduces MMT and tumor progression, underscoring its therapeutic potential [57].

Targeting CAFs presents significant challenges due to their heterogeneity and plasticity. Strategies include preventing fibroblast-to-CAF conversion, disrupting CAF-cancer signaling pathways, and reprogramming tumor-promoting CAFs into tumor-suppressive phenotypes. However, the dual functionality of CAFs complicates therapeutic efforts, as some CAF subsets may protect against tumor progression [58].

Despite these challenges, understanding CAF heterogeneity and their interactions within the TME could pave the way for innovative therapies, improving outcomes for BC patients.

## Tumor-associated macrophages (TAMs)

TAMs are key infiltrating immune cells in the inflammatory microenvironment of malignant tumors, including BC. The infiltration and polarization status of TAMs shape distinct immune microenvironments, which hold predictive significance for survival outcomes, benefits from adjuvant chemotherapy, and sensitivity to PD-L1 blockade therapy, especially in MIBC [59] (Table 1).

TAMs are classified into two functionally distinct groups based on their polarization states: M1 and M2 macrophages. Their hallmark feature is plasticity, the ability to transition between phenotypes in response to changes within the TME [60]. M1 macrophages activate the adaptive immune system, with markers such as inducible nitric oxide synthase (iNOS), ROS, and IL-12. They primarily destroy target cells and support anti-tumor immunity. In contrast, M2 macrophages contribute to angiogenesis, tumor progression, and tissue remodeling, with key surface markers including CD68 and CD163 [61].

Regarding metabolism, M1-TAMs predominantly rely on aerobic glycolysis, while M2-TAMs utilize anaerobic metabolism and secrete lactic acid. This lactic acid further promotes polarization toward the M2 phenotype, which supports tumor progression. M2-TAMs also maintain a strongly immunosuppressive TME by secreting suppressive cytokines, reprogramming immune metabolism, and regulating immune checkpoint signaling pathways [62–64].

TAMs are a predominant immune cell type within the TME, often contributing to immune evasion mechanisms that result in reduced efficacy of ICIs. TAMs frequently express immune checkpoint molecules such as PD-1, PD-L1, and others (e.g., CD39, CD73, and VISTA) that inhibit T cell activation and function [65]. PD-1 expression on TAMs correlates with a reduced M1 phenotype and increased polarization toward the immunosuppressive M2 phenotype. This polarization inhibits anti-tumor immunity and promotes tumor growth. M2-like TAMs secrete IL-10, TGF-β, and other cytokines that suppress T cell proliferation and promote Treg activity. These cytokines enhance the immunosuppressive nature of the TME and contribute to resistance against ICIs.

TAMs enhance PD-L1 expression via tumor-derived factors such as IL-6, granulocyte-macrophage colony-stimulating factor (GM-CSF), and hypoxia-induced hypoxia-inducible factor 1α (HIF-1α) signaling. PD-L1 expressed on TAMs is more closely correlated with resistance to ICIs than PD-L1 on tumor cells, particularly in hepatocellular carcinoma and breast cancer. TAMs suppress the infiltration of CD8^+^ T cells into the tumor and downregulate T cell receptor signaling through direct interaction via immune checkpoint molecules like PD-1/PD-L1. This inhibits cytotoxic T cell-mediated tumor cell killing. Furthermore, TAMs overexpress signal regulatory protein-α (SIRPα), which interacts with the “don’t eat me” signal CD47 on tumor cells. This suppresses macrophage-mediated phagocytosis and facilitates tumor immune evasion [66, 67].

To overcome these challenges, strategies targeting TAMs in combination with PD-1/PD-L1 inhibitors have been proposed. Reprogramming TAMs to an M1-like phenotype is a promising approach. Hydroxychloroquine polarizes TAMs from an M2 to an M1 phenotype, enhancing pro-inflammatory cytokine release and promoting anti-tumor immunity. When combined with PD-1 inhibitors, this approach can amplify CD8^+^ T cell-mediated cytotoxicity [68]. IL-2 therapy induces transcriptional reprogramming of TAMs, increasing the expression of M1 markers (e.g., IL-12, CD86) while suppressing M2-associated genes. Combination therapy with IL-2 and PD-1 inhibitors improves T cell activation and tumor vascular normalization.

Targeting TAM recruitment by blocking the CCL2-CCR2 axis with CCR2 inhibitors reduces immunosuppression and enhances the efficacy of PD-1/PD-L1 inhibitors. Clinical trials combining CCR2 inhibitors with nivolumab are currently underway [60, 69]. Blocking TAM survival pathways using CSF1R inhibitors like PLX3397 or BLZ945 depletes TAM populations by targeting the CSF1-CSF1R signaling axis, which is critical for TAM survival. Preclinical models show enhanced efficacy of PD-1 inhibitors when combined with CSF1R blockers, particularly in hepatocellular [70, 71].

Disrupting “don’t eat me” signals by targeting the CD47-SIRPα axis enhances TAM-mediated phagocytosis of tumor cells. Combining CD47 inhibitors with PD-1/PD-L1 blockade has shown synergistic anti-tumor effects in preclinical studies [72, 73].

Nanoparticle-based therapies have also been developed to target TAMs. Engineered nanoparticles loaded with immunomodulatory drugs or antibodies can specifically target TAMs, reprogramming them into M1-like macrophages or delivering PD-L1 inhibitors directly to the TME. For instance, mannose-modified nanoparticles enriched with metformin (Met@Man-MP) promote TAM reprogramming and improve T cell infiltration [74]. Dual blockade of PD-L1 and PD-L2 on TAMs has shown synergistic effects in preclinical studies, particularly when PD-L2 upregulation compensates for PD-L1 inhibition [75].

Studies have shown that TGF-β secreted by M2-TAMs enhances glycolysis levels in BC and increases PD-L1 expression, thereby promoting immune evasion. These findings were derived from studies using mouse models and organoids generated from tumor tissues [76].

Additionally, CD276, expressed on TAMs, plays a key role in suppressing the immune response against tumors. Researchers revealed that CD276 activates the lysosomal signaling pathway and the transcription factor JUN, regulating the expression of AXL and MerTK, which enhances efferocytosis in TAMs and promotes immune evasion in BC. Depletion of CD276 improved TAM MHC-II expression, increased cytotoxic CD8^+^ T cell infiltration, and reduced tumor growth in a mouse BC model. This study utilized a tumor model induced by *N*-butyl-*N*-(4-hydroxybutyl) nitrosamine in male BC mice, and 93 tumor tissue specimens from BC patients undergoing surgical treatment were analyzed for correlations between CD276 expression and patient survival [77].

Furthermore, macrophages expressing IL-10 are associated with poor prognosis and therapeutic resistance in MIBC. IL-10^+^ TAMs create a robust immunosuppressive TME characterized by anergic CD8^+^ T cells, immature NK cells, and increased immune checkpoint expression. These findings were observed in studies including 128 and 391 patients with MIBC [78].

Another study highlighted the role of low-density lipoprotein receptor-related protein 1 (LRP1) in M2-like macrophage polarization. LRP1 knockdown in BC cells delayed cancer progression both in vivo and in vitro by downregulating M2-like macrophage polarization and suppressing epithelial-to-mesenchymal transition (EMT). Patients with higher LRP1 levels exhibited weaker responses to anti-PD-1 therapy, whereas LRP1 knockdown enhanced the therapeutic effects of anti-PD-1 in mice. These findings were derived from studies using human BC cell lines (5637, T24, TCCSUP, EJ, J82, RT112, and UMUC3), normal human ureteral epithelial cells (SVHUC1), and mouse BC cell lines (MB49) [79].

Li et al. [80] observed that downregulation of E3 ubiquitin ligase SPOP promotes tumor progression and TAM infiltration in BC patients and T24 xenograft models. SPOP deficiency stabilizes STAT3, leading to increased secretion of chemokine CCL2, which induces macrophage chemotaxis and M2 polarization. These studies were conducted in BC cell lines (5637, T24, and SW780), along with the monocytic cell line U937, capable of differentiating into macrophages [80].

### Molecular pathways influencing immune cell interactions in BC

Several significant molecular pathways connect various immune cell types within the TME. These signaling pathways do not operate in isolation; instead, they interact with one another, forming a complex network that governs both tumor cell behavior and the function of surrounding immune cells. Understanding the interactions among these signaling pathways is crucial for developing targeted therapies designed to enhance immune responses and combat cancer.

BC progression and resistance to therapy are influenced by intricate interactions between TAMs, CAFs, and molecular pathways within the TME. CAF activity plays a pivotal role in therapeutic resistance and tumor progression. TGF-β signaling in CAFs promotes stromal remodeling, immune exclusion, and the formation of a physical barrier that prevents T cell infiltration into tumors. This has been demonstrated in studies showing that TGF-β signaling is a key factor in excluding CD8^+^ T cells from the peritumoral stroma. Furthermore, the activation of fibroblasts correlates with poor clinical outcomes and reduced responses to ICIs, emphasizing the importance of targeting this pathway in immunotherapy-resistant tumors [81].

Molecular pathways intrinsic to tumor cells also mediate interactions with immune cell phenotypes and influence therapeutic responses. Wnt-β-catenin signaling has been associated with reduced recruitment of DCs and impaired T cell priming in BC. Gain-of-function alterations in Wnt-β-catenin signaling or MYC, and loss-of-function alterations in PTEN, LKB1, or p53, further impair immune infiltration and contribute to a non-T cell-inflamed TME. Similarly, activation of PPARγ/RXRα signaling suppresses pro-inflammatory cytokines and chemokines, inhibiting CD8^+^ T cell recruitment, and contributing to immunotherapy resistance [82, 83].

The activation of the FGFR3 pathway is another critical mechanism in BC progression. FGFR3-mutated tumors exhibit reduced T cell infiltration despite similar immunotherapy response rates to wild-type tumors. This paradox is attributed to an inverse relationship between FGFR3 mutations and stromal TGF-β signaling. In preclinical models, targeting angiogenesis to normalize tumor vasculature improved immune cell infiltration and oxygen distribution, thus enhancing the immunostimulatory capacity of the TME [84].

**Figure 2:**
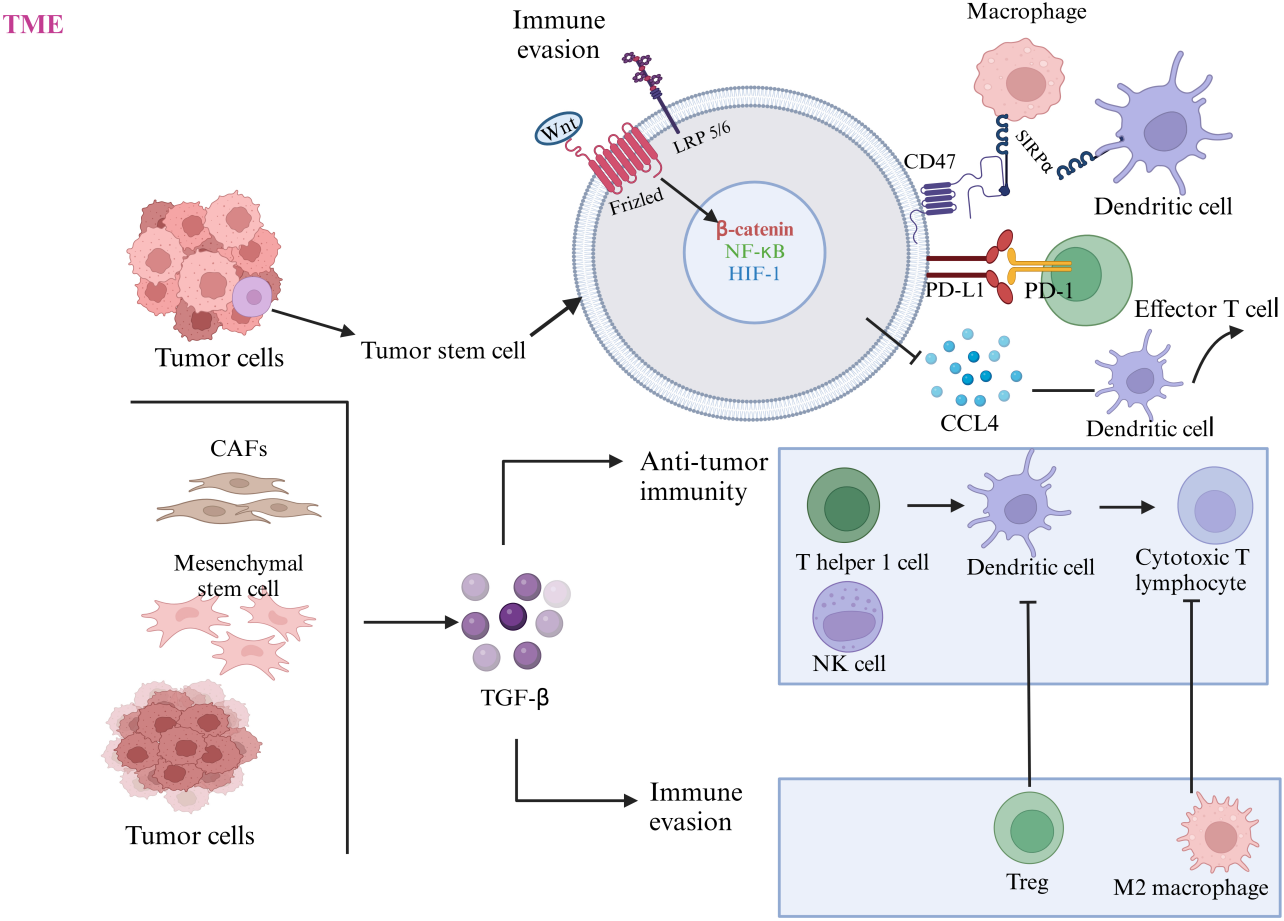
**Role of Wnt and TGF-β pathways in the TME of BC**. TGF-β: transforming growth factor-beta; NK: natural killer; LRP5/6: lipoprotein receptor-related protein 5/6; HIF-1: hypoxia-inducible factor 1; SIRPα: signal regulatory protein-α; PD-1: programmed cell death protein-1; PD-L1: programmed death-ligand 1; TME: tumor microenvironment. Created in BioRender. Balkhi, S. (2025) https://BioRender.com/b19r037

These pathways collectively emphasize the dynamic interplay between immune cell phenotypes, stromal components, and tumor-intrinsic molecular alterations in BC progression and resistance to therapy. The inclusion of diagrams mapping these pathways and their interconnections could enhance the understanding of these concepts and their implications for targeted therapeutic interventions (Figure 2).

Figure 2 highlights the interplay between Wnt and TGF-β signaling pathways in the TME of BC, emphasizing their roles in immune evasion and tumor progression. The Wnt signaling pathway is activated in tumor stem cells through Frizzled and LRP5/6 receptors, leading to the upregulation of β-catenin, NF-κB, and HIF-1. These transcription factors enhance immune evasion by suppressing DC function, promoting the expression of PD-L1, and inhibiting the cytotoxic activity of effector T cells through interactions with PD-1. Additionally, Wnt signaling stimulates the production of CCL4, further disrupting DC-mediated immune responses. The TGF-β pathway, produced by tumor cells, CAFs, and mesenchymal stem cells, drives immune suppression by promoting the expansion of Tregs and M2 macrophages, both of which inhibit anti-tumor immunity. TGF-β also suppresses the activity of T helper 1 cells NK cells, impairing their ability to activate CTLs. Together, these pathways synergize to create an immunosuppressive TME that facilitates tumor growth and metastasis.

### Metabolic pathways regulating TAM function and tumor progression

TAMs display significant metabolic plasticity, allowing them to adapt to the changing and unique metabolic environment within the TME. The interplay between metabolic pathways, particularly fatty acid and glucose metabolism, profoundly influences TAM function, polarization, and their roles in immune suppression and tumor progression.

M1 macrophages, which are classically activated by LPS and IFN-γ, rely primarily on glycolysis and exhibit a pro-inflammatory phenotype, characterized by increased ROS and nitric oxide (NO) production. In contrast, M2 macrophages, which promote tumor growth and immune suppression, rely on oxidative phosphorylation (OXPHOS) and fatty acid oxidation (FAO) as their primary metabolic pathways [85, 86]. The glycolytic enzyme pyruvate kinase isoform M2 (PKM2) plays a crucial role in TAM polarization. Under hypoxic TME conditions, PKM2 dimerization interacts with HIF-1α, driving M2 polarization and promoting immune suppression and tumorigenesis. The metabolic shift in TAMs from glycolysis (M1 phenotype) to OXPHOS and FAO (M2 phenotype) supports tumor growth by suppressing anti-tumoral immune responses and enhancing tumor cell survival [87].

Fatty acid metabolism also plays a central role in TAM function. M2 macrophages show increased fatty acid uptake, accumulation, and oxidation. The uptake of extracellular fatty acids via CD36 and the generation of acetyl-CoA through FAO are essential for M2 polarization and the immune-suppressive functions of TAMs. Dysregulated fatty acid metabolism, including enhanced lipoprotein lipase (LPL) activity and lipid droplet accumulation, provides a continuous supply of fatty acids to TAMs, supporting their tumor-promoting activities [88]. Inhibiting FAO or blocking lipid uptake has been shown to disrupt TAM polarization to the M2 phenotype and impair tumor progression. Both fatty acid and glucose metabolism are intricately linked to the polarization of TAMs and their functional roles in the TME. Shifting the metabolic balance to favor M2 macrophages allows TAMs to suppress anti-tumoral immune responses, promote tumor growth, and contribute to cancer progression, including in BC. Addressing these metabolic pathways presents potential therapeutic opportunities to modulate TAM polarization and enhance anti-tumor immunity.

## Myeloid-derived suppressor cells (MDSCs)

MDSCs are a crucial immune-suppressive myeloid cell type found in the blood and TME of various cancers in humans and murine models. These cells develop during chronic inflammation and contribute to creating an immunosuppressive and proangiogenic environment, significantly impeding the effectiveness of anticancer treatments [89–91] (Table 1).

MDSCs consist of a predominantly immature heterogeneous cell population generated from common hematopoietic progenitor cells, but they are mainly referred to as two distinct subsets: one subset phenotypically resembles polymorphonuclear (PMN) cells, or neutrophils, termed PMN-MDSCs or N-MDSCs; while the other subset is more similar to monocytes, termed M-MDSCs. Both subsets possess inhibitory effects on CD8^+^ cytotoxic and CD4^+^ helper T cells and NK cells via multiple mechanisms, as well as proangiogenic functions [23]. In humans, PMN-MDSCs are characterized by the phenotype CD33^low^CD11b^+^CD14^−^CD15^+^LOX-1^+^HLA-DR^−^, while the M-MDSC counterpart is characterized by CD33^high^CD11b^+^CD14^+^CD15^−^HLA-DR^−/low^ [92].

However, many critical aspects remain regarding the specific markers of the two main MDSC subpopulations and the precise identification of these cells distinct from their neutrophil and monocyte counterparts in the blood and tumor tissues [89]. Among the different mechanisms of immune suppression exerted by MDSCs are the production of reactive oxygen and nitrogen species (ROS and RNS), leading to cell death and/or inhibition of cell proliferation, amino acid metabolism (e.g., arginine and tryptophan), leading to cell exhaustion and/or apoptosis, expression of immune checkpoint ligands like PD-L1 and B7, apoptosis-inducing FasL, and the induction of Tregs and M2-TAMs [93, 94].

Besides their multifaceted immunosuppressive role, MDSCs also play a crucial role in tumor angiogenesis, cancer cell invasion, and metastasis. The angiogenic role of MDSCs derives from their ability to release several proangiogenic soluble factors such as MMP9, TGF-β, bFGF, and VEGF [95].

All these factors sustain the proliferation of aberrant neovessels and facilitate tumor cell extravasation. Of note, MDSCs can also regulate and promote the formation of the pre-metastatic niche by conferring a favorable microenvironment for tumor seeding and are implicated in limiting the effects of cancer therapies, particularly immunotherapies [96–98]. In BC, the number of blood PMN-MDSCs but not M-MDSCs in BC patients was increased compared to healthy individuals [99].

MDSCs infiltrating BC comprise both subsets, and PMN-MDSCs release several proinflammatory chemokines and cytokines, including IL-6, CXCL8, CCL2, CCL3, CCL4, and G-CSF. CD15^+^CD33^low^ MDSCs from BC patients were responsible for the inhibition of CD4^+^ T-cell proliferation [100].

Tumor-infiltrating CD11b myeloid cells consisted of two major MDSC subsets, and the CD11b^+^HLA-DR^+^ cell subset, which represented about 60–70% of all tumor-infiltrated myeloid cells, was highly heterogeneous, comprising a mixture of MDSCs, TAMs, and immature DCs [99].

Several articles have found significant associations between MDSC accumulation, tumor stage progression, and poor prognosis in BC patients [100, 101].

Of note, Zhang et al. [100] identified CXCL2/MIF-CXCR2 signaling as a crucial mechanism by which tumor cells induce MDSC accumulation and expansion in BC. Enhanced expression of CXCL2 and MIF, together with higher numbers of CXCR2^+^CD33^+^ MDSCs, was found in BC tissues, and these increases were significantly associated with advanced disease stages and poor patient prognosis. BC patients had significantly lower percentages of CD14^+^CD15^−^CD33^+^CD11b^+^HLA-DR^−^ M-MDSCs and significantly greater percentages of CD14^−^CD15^+^CD33^+^CD11b^+^HLA-DR^−^ PMN-MDSCs [100].

## BC stem cells

Under physiological conditions, stem cells are located in the basal cell layer of the urothelium, where they maintain homeostasis, renewal, and integrity of the urothelium after injury [102]. CSCs are a subpopulation of tumor cells that possess stem-like properties, including self-renewal and the ability to differentiate into multiple cancer lineages [103] (Table 1). CSCs reside in niche environments within the TME, particularly in oxygen-deprived regions. Hypoxia-related gene expression in these regions promotes the maintenance of CSCs’ stem-like characteristics [104]. CSC expression has been identified in several human solid tumors, including breast, ovarian, lung, and prostate cancers, where it is significantly associated with metastasis-free survival and worse clinical outcomes. It is well-established that CSCs may originate from normal stem cells that acquire genetic mutations through complex mechanisms.

In BC, studies have shown that CSCs express basal cell markers such as CD44, OCT4, and cytokeratin 5 (CK5), suggesting that bladder CSCs may originate from the basal layer, potentially from urothelial stem cells. These findings were derived using a xenograft model for human BC [105].

Bladder CSCs express several markers related to self-renewal, proliferation, and tumor progression. Examples include CD44 and CD133 (self-renewal), SRY homology box 2 (SOX2) (proliferation and progression), and markers of tumor initiation, such as histone 3 lysine 4 methyl transferase 2 (MLL2) and G protein-coupled receptor family C group 5 member A. Among these, OCT4 is a particularly significant marker. OCT4, a key regulator of self-renewal in embryonic stem cells, exhibits high expression in human BC and is associated with tumor aggressiveness and progression [106].

Studies comparing tumor samples and non-tumor bladder tissues from healthy individuals revealed lower expression of OCT4 in non-tumor tissues. Additionally, a higher molecular weight of OCT4 was observed in bladder tumors compared to non-tumor cells, suggesting that differential posttranslational modifications of OCT4 may play a role in bladder carcinogenesis. However, this hypothesis warrants further investigation [106].

Another important marker, SOX2, a well-known transcription factor for stem cells, is upregulated in both mouse and human BC. SOX2 expression has been observed in pre-neoplastic bladder tumors but is absent in normal urothelial cells [107]. Depletion of SOX2-expressing cells in invasive BC models led to enhanced tumor regression, underscoring the essential role of SOX2-positive cells in BC progression. These observations were based on mouse models and 22 human BC samples [107].

STAT3 has been identified as another key regulator of normal and CSCs [108]. Wang et al. [109] demonstrated that upregulation of STAT3, through increased phosphorylation, enhances tumor invasiveness and cancer stem-like properties in BC. Knockdown of STAT3 expression and inhibition of its activation significantly reduced the motility, anchorage, and proliferative abilities of BC cells. Moreover, STAT3 downregulation led to a loss of tumor-forming ability in immunodeficient mice [109].

CSCs are also characterized by mutations in genes involved in tumor progression and EMT, such as those associated with the Wnt/β-catenin and PI3K/Akt pathways [110]. Given the consistent presence of CSCs in the TME and their involvement in tumor progression, targeting bladder CSCs represents a promising therapeutic approach. However, a major challenge lies in the difficulty of directly targeting CSCs due to their similarity to normal stem cells [111].

In an effort to address this, Miyata et al. [112] developed a CSC/cancer-initiating cell (CIC) model using a human BC cell line (UM-UC-3 cells). Through this model, they identified antigenic peptides, such as GRIK2-derived peptides, which could serve as potential targets for bladder CSC/CIC-targeted immunotherapy [112].

## T cells

In the TME of BC, T cells, specifically cytotoxic CD8^+^ T cells, play a crucial role in immune responses against the tumor. These T cells have the ability to directly attack cancer cells, making them a central focus of cancer immunotherapy research (Table 1). Studies have shown that greater infiltration of CD8^+^ T cells in the TME is associated with more favorable clinical outcomes, such as improved overall survival, disease-free survival, and disease-specific survival, in MIBC and NMIBC. The effectiveness of cytotoxic T cells is influenced by immune checkpoints, particularly the PD-1/PD-L1 axis. The ICI antibodies targeting PD-1 and PD-L1 are a cornerstone of immunotherapy for advanced BC. PD-1 is a receptor expressed on T cells, and its interaction with PD-L1 or PD-L2 leads to suppression of T cell activity, promoting immune evasion by the tumor. In the context of the TME, PD-1 expression is often associated with T cell exhaustion, a state where T cells lose their ability to effectively respond to chronic antigen stimulation, such as in the presence of a persistent tumor. Research has shown a strong correlation between the infiltration of both CD8^+^ T cells and PD-1^+^ T cells in the stromal areas of the TME. This suggests that immune exhaustion within the TME is a significant factor limiting the effectiveness of the immune response against the tumor. As a result, targeting the PD-1/PD-L1 pathway with ICI has become a key strategy in reactivating exhausted T cells and enhancing their cytotoxic activity. While PD-L1 expression in BC has been linked to advanced tumor stage and poor overall survival, the results of studies investigating this relationship are somewhat conflicting. Variations in patient characteristics, treatment regimens, and the definition of PD-L1 positivity contribute to these discrepancies. Therefore, while PD-L1 expression holds promise as a prognostic marker, its clinical implications need to be interpreted with caution [113–116].

CD4^+^ T cells have a critical role in TME of BC, and their functions in anti-tumor immunity are increasingly recognized as more versatile than previously thought. Historically, CD4^+^ T cells were considered primarily as helpers for CD8^+^ T cells, aiding in their activation and support through cytokine production and direct interactions with DCs. Their primary role was thought to be indirect, either through promoting CD8^+^ T cell-mediated tumor killing via T-helper 1 (Th1) or suppressing immune responses through Tregs. However, emerging evidence now highlights that CD4^+^ T cells can directly engage in tumor cell killing, marking them as potential key players in anti-tumor immunity.

Recent studies have revealed the presence of cytotoxic CD4^+^ T cells in the TME of BC. These cytotoxic CD4^+^ T cells express granzyme K and granzyme B and exhibit the ability to kill autologous tumor cells in an MHC-II-dependent fashion, suggesting a direct role in tumor destruction. This discovery challenges the traditional view of CD4^+^ T cells as mere helpers and places them at the forefront of immune-mediated tumor killing in BC. Interestingly, these cytotoxic CD4^+^ T cells are found to be susceptible to inhibition by autologous Treg populations, which points to a complex balance between effector and Tregs within the TME [117].

Furthermore, CD4^+^ T cells have been shown to influence the differentiation, survival, and functionality of CD8^+^ T cells, which are the primary effectors of tumor killing. CD4^+^ T cell help is essential for maintaining the effector-memory phenotype of CD8^+^ T cells, preventing their exhaustion in the TME [118, 119]. Without CD4^+^ T cell help, CD8^+^ T cells tend to become exhausted, which limits their anti-tumor effectiveness. The plasticity of CD4^+^ T cells is also notable; mature CD4^+^ T cells can adopt a cytotoxic phenotype under certain conditions, exhibiting MHC-II-restricted cytotoxicity, which is essential for their direct role in killing tumor cells [120].

The role of cytotoxic CD4^+^ T cells is not unique to BC. Studies in other cancers, such as colon cancer and melanoma, have also identified similar cytotoxic CD4^+^ T cell populations. For instance, tumor-reactive CD4^+^ T cells can induce tumor rejection in mouse models when transplanted and treated with anti-CTLA-4, demonstrating that cytotoxic CD4^+^ T cells can directly target and destroy tumor cells. In BC, the presence of cytotoxic CD4^+^ T cells has been associated with improved responses to PD-L1 inhibitors, suggesting their potential as a predictive biomarker for treatment efficacy. These findings reinforce the notion that CD4^+^ T cells are not only essential for orchestrating immune responses but can also actively participate in the elimination of cancer cells [121, 122].

The discovery of cytotoxic CD4^+^ T cells in BC and their role in response to PD-L1 inhibition is significant because it highlights a previously underappreciated aspect of immune-mediated tumor killing. In the IMvigor 210 trial, which examined the response of metastatic BC patients to the PD-L1 inhibitor atezolizumab, the presence of cytotoxic CD4+ T cells in inflamed TMEs was linked to a higher likelihood of response to therapy. This suggests that the immune context of the tumor, including the balance of cytotoxic CD4^+^ T cells, may influence the effectiveness of immunotherapies. In contrast, tumors with an immune-excluded or immune-desert phenotype did not exhibit the same association, underscoring the importance of a robust immune microenvironment for successful immunotherapy [123].

Overall, CD4^+^ T cells in the TME of BC are emerging as key actors not only in supporting CD8^+^ T cell responses but also in directly engaging in tumor cell killing. The recent discovery of cytotoxic CD4^+^ T cells challenges the traditional view of CD4^+^ T cells as mere helpers and suggests new avenues for enhancing anti-tumor immunity. These findings could have significant implications for the development of immunotherapies, particularly those involving ICIs, and for identifying new targets for cancer treatment.

### Regulatory T cells (Tregs)

Tregs play a central role in immune regulation and are involved in a wide range of diseases, from autoimmunity to cancer [124]. In TME, Tregs are a critical component, with their importance stemming from their functional and phenotypic heterogeneity [125] (Table 1).

Tregs are characterized by high expression of the interleukin-2 receptor alpha chain (IL-2Rα, CD25) and the transcription factor FOXP3, both of which are essential for their regulatory functions [126]. FOXP3 expression is particularly crucial for Treg suppressive activity, as mutations in the FOXP3 gene can lead to severe autoimmune diseases in humans [127].

Tregs have traditionally been recognized as suppressors of anti-tumor immune responses, often contributing to poor outcomes in cancer patients. However, recent studies have highlighted their paradoxical roles in promoting tumor suppression in specific cancer subtypes, including BC [128]. Winerdal et al. [129] observed an inverse correlation between Treg frequency and MMP2 expression, a pro-invasive factor activated by tumor-promoting inflammation. In this study, higher Treg expression was associated with improved survival in BC patients, likely due to reduced MMP2 expression. Notably, MMP expression tends to be elevated in invasive stages of BC [130]. Primary tumors can induce MMP2 expression in tissue macrophages, promoting metastasis and underscoring the role of macrophage-derived MMPs in metastatic progression [128]. This study involved peripheral blood and tumor tissue samples from patients, including 28 with MIBC and 18 with NMIBC [129].

In contrast, other research utilizing scRNA-seq has revealed specific subsets of Tregs in BC that exhibit elevated expression of the T-cell immunoreceptor with immunoglobulin and immunoreceptor tyrosine-based inhibition motif domain (TIGIT) as well as IL-32. These TIGIT^+^ Tregs were found to suppress anti-tumor immune responses, and IL-32 was shown to promote BC cell migration and invasion, contributing to metastasis. Targeting TIGIT has demonstrated potential in reinstating anti-tumor immunity and inhibiting BC metastasis [131].

The paradoxical roles of Tregs have also been explored in other tumor subtypes. Anz et al. [132] observed that in medullary breast cancer, a subtype characterized by prominent lymphocytic infiltration, a high ratio of tumor-infiltrating intraepithelial Tregs was associated with a favorable prognosis. This suggests that in this subtype, Tregs may contribute to an anti-tumor immune environment [132].

Another study reported an increased risk of death with higher numbers of TAMs, while a higher frequency of Tregs was associated with a decreased risk of death [133]. This analysis used 145 formalin-fixed, paraffin-embedded tissue samples from MIBC patients. These findings highlight the complex nature of Treg functions within the TME, which can vary significantly across different cancer subtypes and stages. Understanding the subtle roles of Tregs is crucial for advancing targeted immunotherapies that may either enhance their tumor-suppressive capabilities or mitigate their tumor-promoting effects, depending on the cancer context.

The relationship between poor responses to intravesical BCG therapy and Treg/TAM populations in the TME has also been explored. A study involving 71 patients demonstrated that high numbers of Tregs and TAMs were predictive of poor prognosis, suggesting a correlation between BCG resistance and the presence of these immune cells in the TME [134]. In addition, detailed proteomic analyses have provided insights into the role of Tregs in sentinel lymph nodes of BC patients. Researchers observed that growth and immune signaling pathways were upregulated in sentinel lymph node-resident Tregs [135]. Cytokine IL-16 was identified as a central component of the sentinel lymph node-Treg signaling network. Tumor-released factors were shown to activate caspase-3, enhancing the processing of IL-16 into its bioactive forms, further reinforcing the suppressive capacity of Tregs in BC [136].

## B cells

B cells, traditionally recognized for their role in antibody production, have emerged as significant players within the TME of BC, influencing tumor progression, immune responses, and therapeutic outcomes. B cells within the TME exhibit dual roles, acting as both promoters and inhibitors of tumor progression, depending on their subtypes, interactions, and the specific characteristics of the tumor (Table 1).

In NMIBC, the presence and function of B cells within the TME have been linked to patient responses to BCG immunotherapy. Yolmo et al. [134] identified a subset of atypical B cells that not only promote cancer progression but are also associated with poor responses to BCG treatment. These atypical B cells contribute to an immunosuppressive milieu, hindering the efficacy of immunotherapy in NMIBC patients [137].

In contrast, in MIBC, B cells can exhibit anti-tumor properties. Research utilizing single-cell sequencing of B and T cells derived from BC patients has revealed the heterogeneity of B cell subpopulations within the MIBC TME. Notably, the formation of tertiary lymphoid structures (TLSs)—organized clusters of immune cells, including B cells—has been correlated with enhanced patient outcomes. TLSs facilitate the recruitment and activation of immune cells, thereby boosting anti-tumor immunity. The presence of TLSs and specific B cell subsets within the TME may serve as predictive factors for patient prognosis and response to immunotherapies [135].

However, the role of B cells in BC is complex and context-dependent. Some studies using human samples have suggested that specific expressions of B cell-related genes are associated with tumor progression and metastasis. For instance, the chemokine CXCL13, which is involved in B cell recruitment, has been shown to be upregulated in high-grade tumors, particularly in female patients. This upregulation correlates with increased infiltration of M2-like TAMs and poorer clinical outcomes [138].

Moreover, the influence of B cells within the TME on tumor behavior can be further complicated by their interactions with other immune cells and signaling pathways. A study by Ou et al. [139], using human BC tissue samples and mouse xenografted tumors, indicated that recruited B cells could enhance the invasion of BC cells by amplifying IL-8 and androgen receptor (AR) signaling pathways. This amplification results in the upregulation of genes associated with metastasis, such as MMP1 and MMP13, suggesting that, under certain conditions, B cells can modify the TME in a manner that favors tumor progression [139].

Another study by Zhou et al. [140] examined the role of Bregs and associated genes in BC progression and response to immunotherapy.

They observed that IL-10 producing CD19^+^ Bregs can suppress anti-tumor immunity, promoting tumor progression. Additionally, the study identified specific Breg-related genes involved in regulating immune responses within the TME. The findings suggest that targeting Bregs and their associated genes could enhance the effectiveness of immunotherapy in BC patients. By modulating the activity of Bregs, it may be possible to restore anti-tumor immunity and improve patient outcomes.

Comprehending the intricate roles of B cells in BC is essential for advancing targeted treatments and improving patient outcomes.

## Natural killer cells

NK cells are another key group of cells within the TME, serving as important components of the innate immune response by directly killing tumor cells. These cells are antigen-independent and MHC-unrestricted, which enables them to recognize non-self-cells [141] (Table 1).

The effector functions of NK cells are regulated by a balance of signals derived from the interaction between activating and inhibitory receptors on NK cells and their respective ligands on cancer cells [142].

Two major human blood NK subgroups have been characterized based on the surface expression of the neural cell adhesion molecule (NCAM/CD56) and the Fc-gamma-receptor III (CD16) molecule: CD56^dim^CD16^+^ NK cells, which comprise about 90–95% of NK cells, are endowed with cytotoxic activities via perforin and granzyme release and mediate antibody-dependent cellular cytotoxicity (ADCC) via CD16, and CD56^bright^CD16^low/−^ NK cells, 5–10% of circulating NK cells, are more immature lymphoid cells that can rapidly expand and release various inflammatory factors, including IFN-γ, tumor necrosis factor-α, GM-CSF, or regulatory soluble factors, such as interleukins IL-10, IL-13, depending on the stimulatory signals produced in the microenvironment. The CD56^bright^CD16^low/−^ NK cell subset can acquire the mature CD56^dim^CD16^+^ NK cell phenotype, through the downregulation of the CD56 molecule and the acquisition of the CD16 receptor and other surface molecules, such as killer cell immunoglobulin-like receptors (KIRs) [143].

NK cell activity is enhanced by certain cytokines, such as IFN-γ and IL-2 [144]. Several animal studies, including human xenograft models and pilot clinical studies, have demonstrated that IL-2-activated NK cells could be effective against solid tissue metastases [145]. Numerous studies have indicated that the presence of NK cells is associated with better outcomes in BC [146, 147].

However, in several solid tumors or in malignant pleural effusions, it has also been shown that NK cells could have a dual role in both the antitumor response and promoting angiogenesis [148].

Angiogenesis is a hallmark of malignant tumors, and together with hypoxia, TGF-β could be an important factor driving vascular network enhancement as well as the exhaustion of anti-tumor cytotoxic cells and NK cell dysfunction [149–153].

These two NK cell subsets can act quickly as effective cytolytic cells against tumor, infected, or stressed cells without MHC priming via integration of activating and inhibitory signal ligands on surface receptors of NK cells. Interestingly, another NK subset has been found in the decidua, known as the decidual NK (dNK) cell, which comprises about 50% of the total decidual lymphocytes. Notably, dNK cells have a tolerogenic and proangiogenic phenotype, identified as CD56^superbright^CD16^−^VEGF^high^PlGF^high^CXCL8^+^. While in the developing decidua, dNK cells are fundamental innate cells driving the genesis of the spiral artery during embryonic development [154, 155].

In the context of some solid tumors, NK cells could be polarized to a CD56^bright^CD16^−^ phenotype that can act as pro-tumor, proangiogenic cells [156].

Tumor decidual-like NK cells in NSCLC patients have been reported to functionally foster angiogenesis in vitro via soluble factors [157].

These cells were able to induce endothelial cell chemotaxis and formation of capillary-like structures in vitro. Of note, TGF-β1 stimulation of peripheral blood mononuclear cells of healthy subjects induced production of proangiogenic factors, like VEGF and PlGF in CD56^+^CD16^−^ NK cells [158].

Wong et al. [158], through in vitro studies and using also mice models, highlighted that BCs are rich in TGF-β, which triggers downregulation of CD16 and consequently inhibits NK cell-mediated ADCC. By using TGF-β inhibitors, it is possible to restore NK cell tumoricidal activity in BC [158].

Additionally, some researchers [159] have found that both TME-specific TGF-β and metabolic dysfunctions, such as hypoxia, can directly suppress the effector functions of NK cells. Notably, an oxygen-dependent reduction in signaling through SLAMF6 was responsible for diminished NK cell function. These studies were conducted in vitro. Thus, modulating SLAMF6 signaling, particularly enhancing it, may represent a promising strategy to improve NK cell effector functions in BC [159].

However, in solid tumors or in malignant pleural effusions, it has also been shown that NK cells could be anergic and polarized into a functional pro-angiogenic state, resembling M2-TAMs or able to recruit and expand pro-tumor myeloid cells [160–164].

## Polymorphonuclear neutrophils

Circulating human PMNs are a large group of innate immune cells with a very short half-life (6–10 h) after their release from the bone marrow. They then undergo rapid apoptosis and are ultimately eliminated by the liver and spleen [165–167]. Their main functions include the phagocytosis of microorganisms, generation of reactive oxygen intermediates, and the release of lytic enzymes with antimicrobial potential [168] (Table 1).

PMNs also contain a large amount of VEGF, which is involved in angiogenesis [169]. The role of PMNs in antitumoral immune responses is controversial [170].

Under inflammatory conditions, PMNs can promote tumor growth and progression in response to inflammatory stimuli. Some studies have primarily focused on the role of neutrophils in promoting tumor metastasis through the generation of neutrophil extracellular traps (NETs) [171].

NETs are extracellular structures composed mainly of DNA, which is the primary component, and are selectively degraded by plasma DNase I. These traps also contain histones and cytoplasmic proteins, including calprotectin, myeloperoxidase, and elastase [172, 173].

NETs have dual functions. On one hand, they support tumor growth, angiogenesis, and invasiveness by favoring the extravasation of tumor cells into the bloodstream, thus promoting metastasis. On the other hand, NETs can reduce the activation threshold of T cells and promote an adaptive immune response, which may help eliminate cancer cells through the release of cytotoxic substances [174, 175].

The formation of NETs is a process known as NETosis, during which PMNs release these extracellular traps. Herranz et al. [174] highlighted a significant increase in blood neutrophil counts and NET markers in the plasma of BC patients compared to healthy controls. They also observed a decrease in DNase I activity in the plasma of BC patients, which could explain the increase in NETs found in their plasma.

Despite the potential negative aspects, especially in the context of therapeutic approaches, PMNs can exert important antitumor functions. Several in vivo studies using murine models and in vitro studies report an anti-tumor role of PMNs, especially after intravesical BCG instillation. Suttmann et al. [168] demonstrated that BCG-stimulated PMNs, as a significant group of immunoregulatory cells, can orchestrate T-cell chemotaxis to the bladder during BCG immunotherapy. Using functional chemotaxis assays in mice, they showed that activated PMNs induce monocyte migration and utilize the accessory function of these immune cells to attract T cells.

Further studies showed that BC cells rapidly recruit neutrophils through CXCL8 [176]. These studies also observed that PMNs play a dual role in BC, including the release of hepatocyte growth factor and the formation of a CCL3^high^PD-L1^high^ super-immunosuppressive subset. In particular, c-Fos, a mediator that upregulates CXCL8 transcription, was found to be relevant in BC cells due to its role in the positive feedback loop of neutrophil recruitment [176].

Despite their potential for antitumor activity, increased neutrophil infiltration has been correlated with poor prognosis in BC. Gunes et al. [176] discovered that plasma from BC patients showed an overexpression of three neutrophil proteins: human neutrophil protein-1, -2, and -3 (HNP 1–3). These are subtypes of α-defensins, proteins that trigger leukocyte recruitment, contribute to metastasis, stimulate tumor cell proliferation, and promote angiogenesis.

## Immunotherapy in BC

Immunotherapy has emerged as a first-line treatment for patients with high expression of PD-L1 in BC. Among the most studied approaches are ICIs, particularly PD-1/PD-L1 inhibitors such as atezolizumab, nivolumab, and pembrolizumab, which work to activate T cells in BC [177, 178].

**Figure 3:**
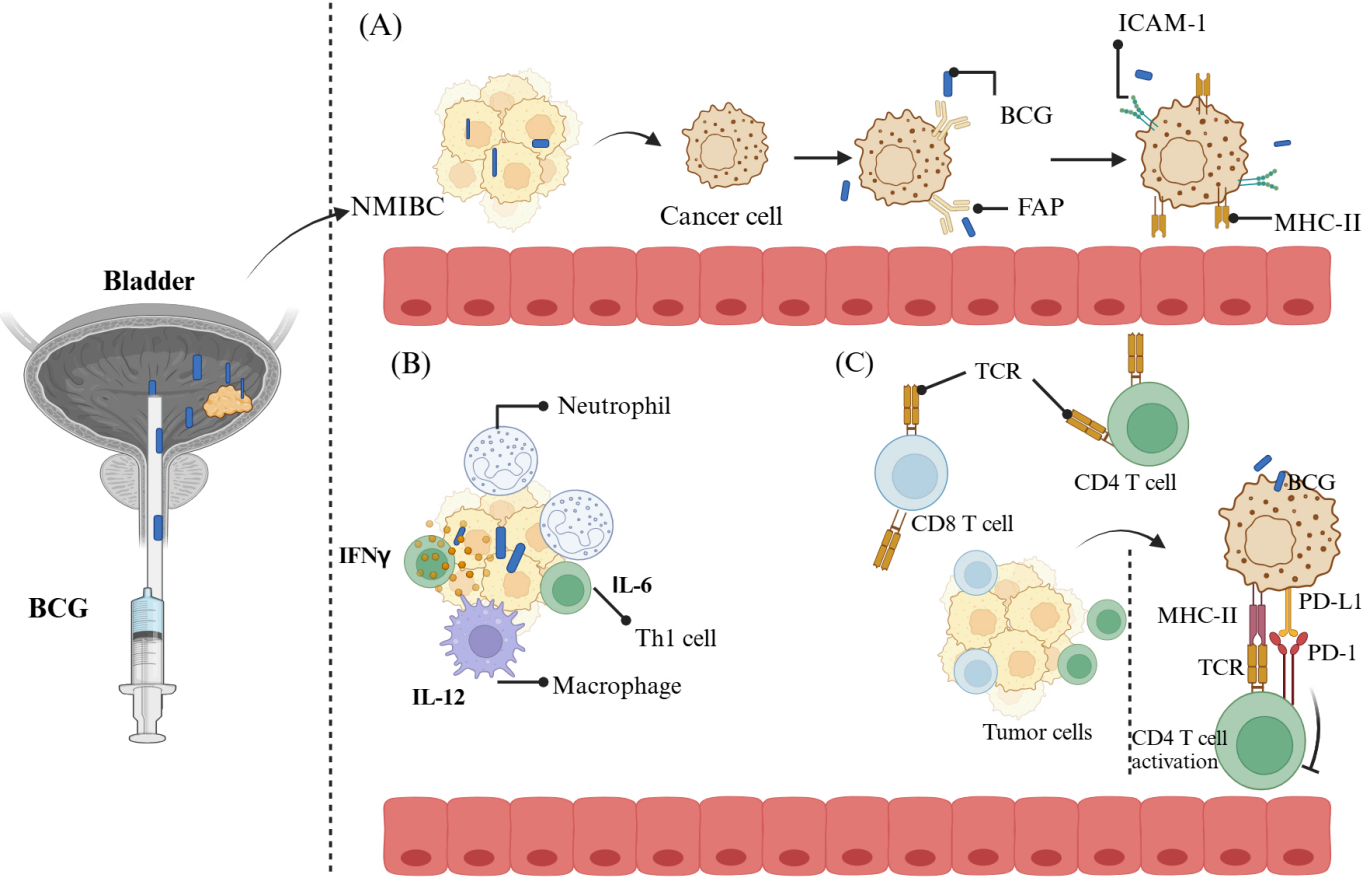
**Immune mechanisms involved in BCG therapy for BC treatment**. (**A**) Following instillation, BCG binds to the urothelium through fibronectin attachment protein (FAP) and is subsequently internalized into tumor cells. This triggers an increase in the expression of MHC-II and ICAM-1 on BC cells; (**B**) BCG facilitates the recruitment of diverse immune cells into the bladder tumor tissue, leading to the release of a variety of inflammatory cytokines and chemokines, which reshape the tumor immune microenvironment; (**C**) BCG stimulates adaptive immune cells, including CD4 and CD8 T cells, enabling them to mediate cytotoxic effects on BC cells. Furthermore, BCG enhances PD-L1 expression on tumors, which may augment the effectiveness of immune checkpoint inhibitors. BCG: Bacillus Calmette-Guérin; NMIBC: non-muscle-invasive bladder cancer; INF-γ: interferon γ; IL-6: interleukin-6; Th1: T-helper 1; TCR: T cell receptor; PD-1: programmed cell death protein-1; PD-L1: programmed death-ligand 1. Created in BioRender. Balkhi, S. (2025) https://BioRender.com/n92t663

Recent advances have seen significant progress in both CAR-T cell therapies and checkpoint inhibitors, with numerous clinical trials underway to evaluate their efficacy and safety. CPI have transformed the management of BC, with key agents including pembrolizumab, nivolumab, atezolizumab, durvalumab, and avelumab, all of which target the PD-1/PD-L1 axis. Pembrolizumab has been FDA-approved for NMIBC in patients unresponsive to BCG therapy and ineligible for radical cystectomy. This approval followed results from the KEYNOTE-057 trial, where 40.6% of patients achieved complete responses [177]. Similarly, nivolumab is under investigation in combination with BCG in trials like CheckMate 7G8, which aim to improve outcomes for high-risk NMIBC patients unresponsive to BCG (NCT04149574). Combination therapies such as pembrolizumab with gemcitabine (NCT04164082) and atezolizumab with BCG (BladderGATE study, NCT04134000) further explore synergies between immune-modulating agents and traditional therapies. As illustrated in Figure 3, BCG therapy not only enhances PD-L1 expression on BC cells but also reshapes the tumor immune microenvironment by recruiting immune cells and activating adaptive immune responses, thereby offering a complementary mechanism to ICIs.

CAR-T cell therapies, though in earlier stages of investigation for BC, hold significant promise. Current research focuses on engineering CAR-T cells to target tumor-specific antigens like HER2 and EpCAM, both highly expressed in BC. Innovative approaches, such as dual-target CAR-T cells, are being developed to overcome challenges like tumor antigen heterogeneity and the immunosuppressive TME. Early-phase clinical trials are underway to evaluate their safety and efficacy in patients with advanced, treatment-resistant disease [179, 180]. Oncolytic viruses represent another exciting avenue in BC immunotherapy. Agents like CG0070, an adenovirus delivering GM-CSF, have shown efficacy in BCG-unresponsive NMIBC patients, with phase II trials demonstrating complete response rates of up to 47% at six months [181]. Similarly, nadofaragene firadenovec, a recombinant adenovirus encoding INF-α2b, demonstrated high-grade recurrence-free survival rates of 72.9% at three months and 43.8% at 12 months in a phase III trial [182].

Immune-targeted gene therapies and cytokine therapies are also advancing. Vicinium, an antibody-drug conjugate (ADC) targeting EpCAM, has demonstrated efficacy in delaying cystectomy in NMIBC patients, with 52% of responders remaining disease-free for 12 months in a phase III trial [183]. Cytokine-based therapies like ALT-803, an IL-15 superagonist, have been studied in combination with BCG, achieving complete responses in all treated patients in a phase Ib trial, with no significant adverse events (NCT02138734). ADCs, such as enfortumab vedotin and sacituzumab govitecan, are being investigated for metastatic BC. These ADCs target nectin-4 and TROP2, respectively, and have demonstrated encouraging outcomes in phases II and III [184, 185] Enfortumab vedotin, in particular, has shown an overall response rate of 44% in patients previously treated with checkpoint inhibitors and platinum-based chemotherapy (NCT03219333).

These therapies illustrate the diversity and potential of immunotherapy approaches for BC, ranging from ICIs to novel modalities like CAR-T cells and oncolytic viruses. Ongoing clinical trials will provide further insights into optimizing these therapies for improved outcomes in both early-stage and advanced BC. The TME plays a crucial role in determining response rates to ICIs, with high levels of antitumor T-cell infiltration and macrophage polarization strongly associated with improved responses. ICIs can reverse immune suppression within the TME, which is often mediated by Tregs, macrophages, and CAFs in BC. Not all individuals exhibit sensitivity to immunotherapy treatments, and the reasons for this lack of response remain inadequately understood. Factors contributing to tumor resistance against antitumor immune responses include irregularities in priming signals, the activation of inhibitory signals through the recruitment of Tregs or immunosuppressive cytokines, and deficiencies in antigens, antigen-presenting cells, and stromal interactions [186].

Fortunately, numerous studies have indicated that combining ICIs with other therapeutic agents may provide a promising strategy to counteract cancer immune evasion. Currently, clinical trials are investigating the effects of combining ICIs with nearly all existing cancer treatments, including radiotherapy, chemotherapy, targeted therapies, local therapies, and other immunotherapies such as adoptive cell therapies [187]. Radiotherapy and chemotherapy are believed to play crucial roles in mitigating immune evasion by tumors. Tumor irradiation significantly induces inflammation at the treatment site, enhances the expression of MHC-I and adhesion molecules, and activates CD8^+^ T cells. A phase I–II clinical trial is currently in progress to evaluate the safety and efficacy of fixed-dose stereotactic body radiotherapy in conjunction with either concurrent or sequential pembrolizumab in patients with BC [188]. Local therapies, including chemotherapy, stimulate the innate immune response, activating adaptive immunity by increasing the availability of tumor antigens from cancer cell death, which enhances T cell responses by creating a more inflammatory environment [189].

Enhancing our understanding of the mechanisms underlying drug resistance in BC is essential for developing effective treatment strategies. CRISPR-based screening has emerged as a significant tool in this pursuit. By facilitating precise gene editing, CRISPR screens can systematically pinpoint genes involved in drug resistance [190] integrated CRISPR screening data with IC_50_ values to uncover genes associated with resistance to trametinib, a MEK inhibitor. Prior research has also validated the effectiveness of this strategy. A comprehensive CRISPR screen across the genome revealed the involvement of the *MSH2* gene in cisplatin resistance in MIBC. Depletion of MSH2 was linked to heightened resistance to cisplatin, suggesting it may serve as a biomarker for predicting chemotherapy response [191].

Studies have also demonstrated that vaccine-induced immune responses can be enhanced by depleting Tregs or inhibiting the production and function of immunosuppressive cytokines. Immunocytokines, which are antibody-cytokine fusion proteins that enhance tumor-specific delivery of immunostimulatory factors such as IL-2, TNF-α, or IL-12, show significant therapeutic potential. Although current clinical trials exploring intravesical IL-12 remain limited, this cytokine is particularly interesting due to its ability to stimulate the immune system and counteract immunosuppression in cancer cells. IL-12 activates macrophages to release IFN-γ, promoting tumor-infiltrating lymphocyte (TIL) activation and reducing the number of Tregs [186].

Another promising strategy is the use of CAR-T therapy, a form of immunotherapy that delivers targeted antitumor effects through genetically modified T cells. CAR-T cells are specifically designed to recognize tumor-associated antigens, which are abundantly expressed in BC cells. Parriott et al. [192] developed PD-1-targeting CAR-T cells to bind the PD-1 receptor ligand expressed in solid tumors, including BC. Their findings revealed that PD-1-CAR-T cells successfully induced tumor cell lysis and achieved long-term tumor-free survival in preclinical mouse models [121]. Currently, clinical studies investigating CAR-T cell therapy for BC are ongoing [192]. In parallel, CAR-NK (CAR-NK) cells are being explored for their direct cytolytic antitumor effects. NK cells hold promise not only for their ability to kill tumor cells but also for shaping the TME and enhancing adaptive immunity. Continued studies will be critical to fully understand and leverage their therapeutic potential [193].

Furthermore, research has focused on targeting CAFs, key components of the TME. Burley et al. [41] investigated the role of the TGF-β pathway in BC and its relationship with CAFs. While targeting CAFs has shown potential in improving patient outcomes, challenges remain due to the complexity of CAF biology and the absence of unique CAF markers. Finally, CSCs represent another therapeutic target in BC. Investigators have summarized key biomarkers and features of CSCs across various cancers, including BC, to outline potential strategies for targeting urological CSCs. These strategies involve critical signaling pathways such as Wnt/β-catenin, cytokines, and angiogenesis factors like VEGF [41].

## Conclusion

Immunotherapy has transformed cancer treatment, offering renewed hope for patients with previously untreatable malignancies. Therapies such as ICIs, immunocytokine treatments, and CAR-T cells have significantly improved outcomes in some cancers. However, their broader application is limited by challenges such as immune resistance and the heterogeneous nature of the TME. The TME, a dynamic ecosystem comprising tumor cells, immune cells, stromal components, and signaling molecules, plays a crucial role in shaping tumor progression and modulating therapeutic responses. In BC, this complexity is further compounded by the presence of immunosuppressive components like Tregs, TAMs, and CSCs, which contribute to immune evasion and therapy resistance.

Recent advances in understanding BC’s immune landscape have highlighted the potential of precision immunotherapy. For instance, the identification of distinct TME immunophenotypes and the development of biomarkers such as the immune cell infiltration score (ICscore) provide critical tools for predicting patient-specific responses to immunotherapy. ICscore has shown promise in stratifying responders to ICIs like anti-PD-L1 and anti-CTLA-4 therapies and correlates negatively with markers of tumor immune escape. These findings emphasize the importance of integrating such biomarkers into clinical decision-making to personalize treatment strategies, optimizing outcomes for each patient.

Numerous recent investigations have utilized the TCGA to examine immune cells in the context of cancer. However, this platform is not without its biases. Despite significant progress in the field, the analysis of transcriptomic data continues to encounter considerable challenges due to both technical and biological biases. Technical biases arise from complications associated with microarray, RNA-seq, and nanopore sequencing technologies, while biological biases are influenced by factors such as tumor heterogeneity and sample purity. Moreover, while bioinformatics analyses provide valuable insights into immune landscapes, the reliance on publicly available datasets such as TCGA and GEO introduces inherent limitations related to sample diversity, data quality, and potential confounding factors. Future studies should incorporate multi-omics approaches, including proteomics and single-cell sequencing, to achieve a more comprehensive understanding of the TME and immune interactions. Additionally, functional validation of computational findings through in vitro and in vivo models remains crucial to translating these insights into clinical applications. It is emphasized the necessity of acknowledging and addressing these biases in transcriptomic data mining to improve the reliability and significance of cancer research.

Personalized immunotherapy approaches tailored to TME characteristics could help overcome challenges associated with immune escape and resistance. Combining immunotherapy with other treatment modalities, such as chemotherapy and radiotherapy, offers additional potential to enhance therapeutic efficacy by targeting both the tumor and its microenvironment. Such integrative strategies could disrupt immunosuppressive niches within the TME, improve immune infiltration, and restore effective anti-tumor responses.

In conclusion, while immunotherapy has achieved remarkable milestones, its success in BC relies on advancing our understanding of TME heterogeneity and leveraging novel biomarkers to guide precision medicine. Combining cutting-edge immunotherapeutic strategies with personalized, TME-informed approaches holds immense promise for overcoming immune resistance, improving patient outcomes, and driving the next era of cancer care.

## Abbreviations

ADC: antibody-drug conjugate
ADCC: antibody-dependent cellular cytotoxicity
BC: bladder cancer
BCG: Bacillus Calmette-Guérin
Bregs: regulatory B cells
CAFs: cancer-associated fibroblasts
CAR-T: chimeric antigen receptor T-cell
CIC: cancer-initiating cell
CSCs: cancer stem cells
CTL: cytotoxic T lymphocyte
CTLA-4: cytotoxic T-lymphocyte-associated protein 4
DCs: dendritic cells
dNK: decidual natural killer
EMT: epithelial-to-mesenchymal transition
FAO: fatty acid oxidation
GM-CSF: granulocyte-macrophage colony-stimulating factor
HIF-1α: hypoxia-inducible factor 1α
iCAF: inflammatory CAF
ICIs: immune checkpoint inhibitors
IFN: interferon
IL-10: interleukin-10
LRP1: lipoprotein receptor-related protein 1
M2-TAMs: M2 tumor-associated macrophages
MDSCs: myeloid-derived suppressor cells
MIBC: muscle-invasive bladder cancer
myCAF: myofibroblastic CAF
NETs: neutrophil extracellular traps
NK: natural killer
NMIBC: non-muscle-invasive bladder cancer
PD-1: programmed cell death protein-1
PD-L1: programmed death-ligand 1
PI3K: phosphoinositide 3-kinase
PMN: polymorphonuclear
ROS: reactive oxygen species
SIRPα: signal regulatory protein-α
SOX2: SRY homology box 2
TAMs: tumor-associated macrophages
TGF-β: transforming growth factor-beta
TIL: tumor-infiltrating lymphocyte
TLSs: tertiary lymphoid structures
TME: tumor microenvironment
Tregs: regulatory T cells
VEGF: vascular endothelial growth factor

## Declarations

### Copyright

© The Author(s) 2025.
